# QSPRmodeler - An open source application for molecular predictive analytics

**DOI:** 10.3389/fbinf.2024.1441024

**Published:** 2024-09-23

**Authors:** Rafał A. Bachorz, Damian Nowak, Marcin Ratajewski

**Affiliations:** ^1^ Institute of Medical Biology, Polish Academy of Sciences, Łódź, Poland; ^2^ Department of Quantum Chemistry, Faculty of Chemistry, Adam Mickiewicz University, Poznań, Poland

**Keywords:** QSPR, machine learning, drug design, biological activity, ADMET

## Abstract

The drug design process can be successfully supported using a variety of *in silico* methods. Some of these are oriented toward molecular property prediction, which is a key step in the early drug discovery stage. Before experimental validation, drug candidates are usually compared with known experimental data. Technically, this can be achieved using machine learning approaches, in which selected experimental data are used to train the predictive models. The proposed Python software is designed for this purpose. It supports the entire workflow of molecular data processing, starting from raw data preparation followed by molecular descriptor creation and machine learning model training. The predictive capabilities of the resulting models were carefully validated internally and externally. These models can be easily applied to new compounds, including within more complex workflows involving generative approaches.

## 1 Introduction

Drug discovery is a process oriented towards the identification and development of biologically active compounds. These molecules are expected to act selectively on certain biological targets, such as enzymes or protein receptors, to influence their biological behavior. A critical element of the drug design process is experimental verification of the ability of a molecule to achieve the desired biological effect. In the early drug discovery stage, this can lead to significant costs because of the large number of drug candidates considered. To avoid this, one can attempt to predict the properties based on existing experimental data. This allows the removal of many compounds and ultimately leaves only the most promising candidates. To achieve this goal, it is necessary to develop a predictive model that properly captures the relationship between the structure of a molecule and its properties. The acronym QSAR represents quantitative structure–activity relationship and relates to a set of techniques capable of predicting the biological activities of compounds based on their structural features. A similar term QSPR, which represents quantitative structure–property relationship, is somewhat more generic and covers any molecular property that can be inferred from the underlying molecular features. The first attempts to QSAR modeling were carried out more than 60 years ago Hansch et al. (1962); Free and Wilson (1964); Hansch and Fujita (1964), and till now are still one of the most important computational tools in the hands of medicinal chemists Tropsha et al. (2003); Murphy (2011). The applications of QSAR/QSPR models are broad and include toxicity Ariëns (1984); Hansch et al. (1995, 1989); Votano (2004); Patlewicz et al. (2008) and metabolism predictions Chohan et al. (2006); Sridhar et al. (2012). QSAR studies are often oriented toward model development that supports virtual screening for promising drug candidates for certain diseases, such as malaria Zhang et al. (2013), schistosomiasis Neves et al. (2016), and influenza Lian et al. (2016). QSAR/QSPR approaches are specifically used in machine learning applications. There are several commercially available programs that cover either partially or completely the QSAR/QSPR workflow. Examples include ADMET Predictor™ from Simulations-Plus (2023), Deep AutoQSAR from Schrödinger Suite Dixon et al. (2016), Biovia Discovery Studio from Dassault Systémes BIOVIA Dassault Systémes (2023), MOE from the Chemical Computing Group (CCG) Chemical Computing Group (2023), VLifeQSAR from VLife Sciences VLife (2023), and Flare™ from Cresset Cresset (2023). All these tools are capable of creating QSPAR/QSAR predictive models based on the entire portfolio of Machine Learning methodologies and various flavors of molecular descriptors/fingerprints. For instance, DeepAutoQSAR provides Deep Neural Network methodologies based on custom implementation of molecular descriptors, allowing for the training and application of state-of-the-art quantitative structure-activity relationship (QSAR) models. The Flare ™ module from Cresset provides a Multilayer Perceptron method and a set of other Machine Learning methodologies supporting the development of consensus regression and classification models. Other vendors provide tools that differ slightly in various aspects; however, their common denominator is the commercial nature of their programs. The main goal of the proposed software is to provide an open-source alternative in the form of Python scripts based exclusively on available open-source cheminformatics and machine learning libraries, which implement a complete QSAR/QSPR workflow. In addition, the functional scope of the proposed QSPRmodeler software is beyond the scope of standard machine learning or cheminformatics libraries that are considered separately. The main novelty of the proposed solution is that it combines these two worlds into a single entity, allowing for the straightforward management of chemical information and efficient extraction of predictive signals. Moreover, the proposed functional design enables the incorporation of new machine learning methodologies, which makes the open-source society a toolset capable of exploring novel predictive approaches in a chemical context.

## 2 Software description

**FIGURE 1:**
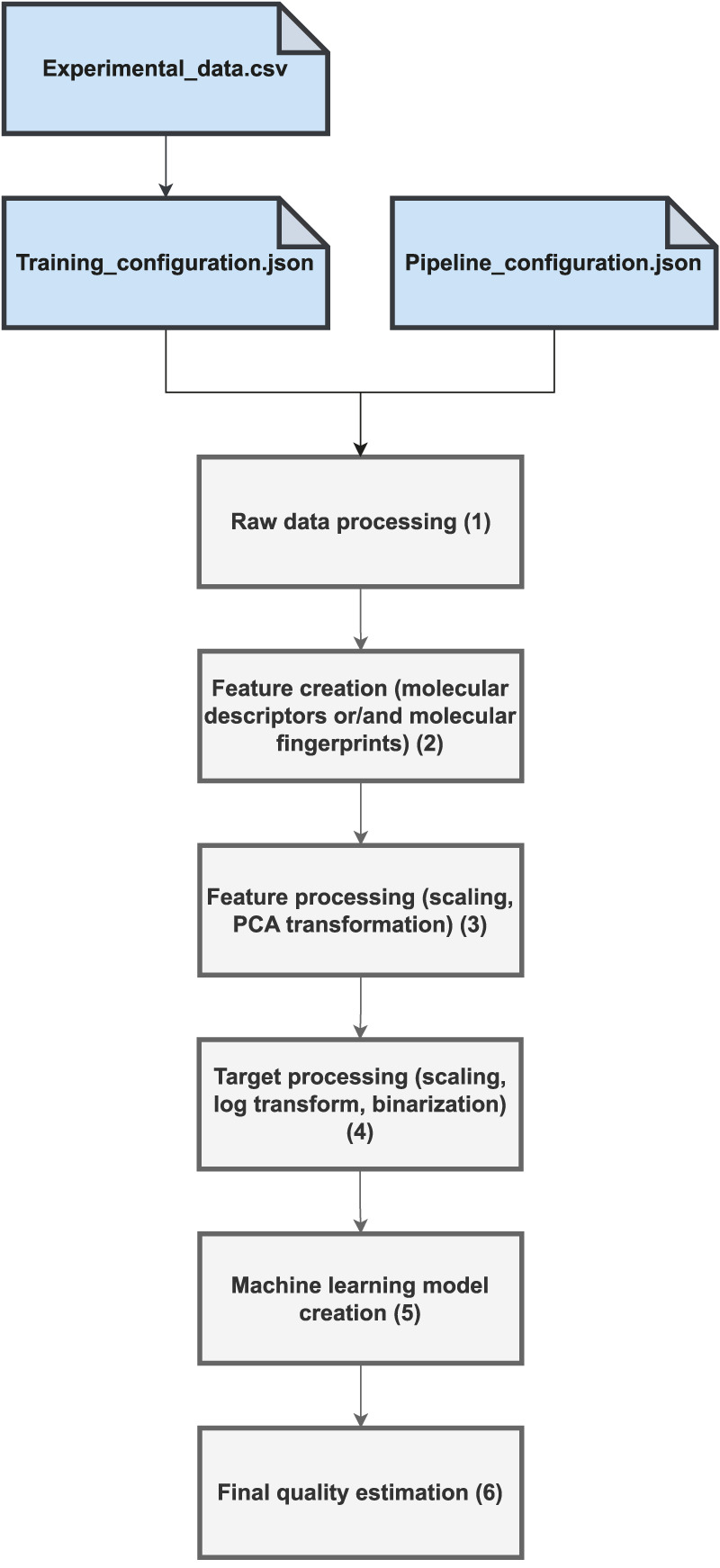
General workflow of the QSPRmodeler program.

The proposed software combines existing Python libraries to cover all the key steps of the QSAR/QSPR modeling process. The workflow is shown in Figure 1. The entire calculation depends on three files: the data file with experimental values in csv form (denoted as Experimental_data.csv), the training configuration (denoted as Training_configuration.json), and the data processing pipeline file (denoted as Pipeline_configuration.json). The incoming data must be prepared in a simple form of a csv file, with the SMILES Weininger (1988) code accompanied by the experimental data. The experimental data are usually IC50 or EC50 values expressed in molar units. Multiple experimental values often exist for the same compound. These values may have been derived from an entirely independent experimental investigation involving qualitatively different biological assays. Thus, an unwanted effect is the potential inconsistency in the experimental endpoints for the same compound. The raw data prepossessing phase (denoted as “1” in Figure 1) measures the level of inconsistency as a standard deviation and removes cases in which it exceeds a certain threshold value, as defined in the Training_configuration.json file (set at the level of 100 nM). For the remaining consistent cases, the chosen aggregation strategy is applied, for example, the arithmetic mean, median, maximum, or minimum function. The resulting dataset is then a simple table associating a certain molecule in the SMILES form Weininger (1988) with single, potentially aggregated, experimental values. In the next step, denoted as “2” in Figure 1, the molecular features are calculated. In particular, various types of molecular fingerprints, such as daylight fingerprints Daylight Chemical Information Systems (2019), atom-pair fingerprints Carhart et al. (1985), topological torsion fingerprints Nilakantan et al. (1987), Morgan fingerprints Rogers and Hahn (2010) and MACCS keys Durant et al. (2002) can be obtained. All these are calculated using the open-source RDKit chemical informatics library Landrum (2022). Selected molecular descriptors can also be incorporated to augment the molecular feature space further. To achieve this, we integrated the Mordreds library Moriwaki et al. (2018) offering an implementation of 1825 molecular descriptors. The next step of the workflow, denoted as “3” in Figure 1 shows the standard data processing steps for molecular descriptors, such as scaling and Principal Component Analysis (PCA) transformation. The latter can be applied to both the molecular fingerprints and descriptors. This step is accomplished using the Scikit-learn library Pedregosa et al. (2011). Step “4” of the workflow takes care of the processing of the target value, in particular logarithm transformation of the numerical value in the case of regression models or target binarization in the case of the binary classifiers. Upon completion of this step, all data are prepared for predictive analytics. The next step, denoted as “5,” applies the chosen machine learning methodology to the prepared, earlier data. Currently, six common predictive model types are available: extreme gradient boosting (XGBoost) Chen and Guestrin (2016), artificial neural networks in the form of multilayer perceptrons Bishop (1995), support vector machines Cortes and Vapnik (1995); Vapnik (1998), random forests Ho (1995), ridges Hoerl and Kennard (1970), and bagging models. With each of these methodologies, a definition of the hyperparameter space is provided. The predictive model creation step involves hyperparameter optimization within the Hyperopt framework Bergstra et al. (2013), which implements the heuristics of the Tree of Parzen Estimators Bergstra et al. (2011). The last step of the workflow, denoted as ”6,” involves final quality measures calculation and model serialization. The final predictive model is stored in a dedicated file together with all the auxiliary information required for the subsequent standalone application in the inference mode. In particular, the entire data-processing pipeline is serialized such that the only information required to perform the prediction is the molecule provided in the form of a SMILES code. The program automatically turns the SMILES representation into a feature space compliant with the model interface and ultimately provides the prediction. A serialized model is an autonomous artifact that is easily integrated into various workflows and molecular predictive use cases. The associated Github repository contains a set of Jupyter notebooks that illustrate how QSPR models can be used in the inference mode, for example, to predict the molecular/biological properties of new compounds. One can imagine multiple, more complex use cases, such as virtual screening of molecular databases, application of unsupervised learning to molecular data, or integration with generative chemistry workflows where models are created that are responsible for criticizing new species against optimized properties. The module is also prone to potential extensions, and an intermediate Python programmer can easily add a new predictive methodology or adopt the provided scripts for particular needs.

## 3 Illustrative examples

### 3.1 Introduction

As illustrative examples, we applied the presented methodology to QSAR modeling of the inhibitory effects of the human androgen receptor (AR) and the activation effects of the pregnane X receptor (PXR) receptor. The former (AR, NR3C4) belongs to the nuclear receptor subfamily 3, a group C nuclear receptor superfamily of proteins Burris et al. (2013). AR acts as a transcription factor that regulates genes important for the development and maintenance of primary and secondary male characteristics Heemers and Tindall (2007). Similar to other nuclear receptors, AR activity is regulated by low-molecular-weight ligands. In the absence of a ligand, the AR resides in the cytoplasm bind to heat shock proteins (HSPs). Upon binding to the ligand, the receptor changes its conformation, homodimerizes, and translocates into the nucleus to regulate AR-dependent genes Prescott and Coetzee (2006). Testosterone and dihydrotestosterone are the endogenous ligands of AR. Under physiological conditions, AR is involved in the development of prostate; however, the disturbed function of these receptor leads to uncontrolled proliferation of prostate cells and the appearance of cancer Lonergan and Tindall (2011), Jernberg et al. (2017). Prostate cancer cells require androgens for survival and proliferation, which is why therapies that use anti-androgens targeting the function of AR are generally effective Kokal et al. (2020).

The second receptor, PXR (NR1I2), regulates xenobiotic metabolism and is involved in the maintenance of liver physiology Cai et al. (2021). This receptor recognizes a wide range of structurally diverse compounds, including endogenous metabolites such as bile acids Staudinger et al. (2001), phthalates Hurst and Waxman (2004), and mycotoxins Ratajewski et al. (2011), and responds to various pharmacologic compounds, including but not limited to rifampicin, dexamethasone, clotrimazole, etoposide, trifluridine, and mycophenolic acid Moore et al. (2000); Ratajewski et al. (2015); Yim et al. (2023). Interactions between pharmacological compounds and PXR are crucial, as PXR recognition can markedly enhance the liver transformation rate of various xenobiotics, leading to potential drug-to-drug interactions Lehmann et al. (1998); Fuhr (2000); Ratajewski et al. (2015).

This encouraged us to use the presented computer-based QSPRmodeler environment by applying machine learning to identify novel chemical structures targeting the ligand-binding domains (LBD) of both receptors. All available experimental values, the IC50 values for AR, and the EC50 values for PXR (half-maximal inhibitory and effective concentrations, respectively, for IC50 and EC50), were retrieved from the ChEMBL 3.3 database Gaulton et al. (2012). The experimental IC50 data for the AR reflected the IC50 measurements of 1575 different chemical species. For the other receptor, we found 1187 entries of EC50 values. Multiple experimental values are often obtained for the same molecule using different biochemical assays. The experimental values obtained for the same molecule cannot always be combined; therefore, we carefully analyzed the available data and excluded doubtful experimental endpoints. The resulting datasets were used to create regression and binary classification models using the QSPRmodeler toolset presented herein. The target value of the regression model was chosen as the negative logarithm of IC50 or EC50, denoted as pIC50 or pEC50, respectively. The classification model was trained on the binarized data, that is, each molecule was assigned to the ACTIVE or INACTIVE class with the class-determining threshold value assumed to be 1000 and 12,000 nM, respectively, for the IC50 values of the AR and EC50 values of the PXR. According to the configured workflows, the models were trained using the XGBoost method within a 5-fold crossvalidation scheme. The space of molecular features was limited to 8 descriptors: SLogP (octanol-water partition coefficient), SMR (molar refractivity), naRing (number of aromatic rings), nHBAcc (number of hydrogen bond acceptors), nHBDon (number of hydrogen bond donors), nRot (number of rotatable bonds), MW (molecular weight), and TopoPSA (topological polar surface area). In addition, the feature space was augmented with two drug-like filters, the Lipinsky rule-of-five and Ghose filters, and 50 most important principal components were calculated based on a 1024-bit long Morgan fingerprint. Thus, each molecule was characterized by a vector of 60 numbers, reflecting its topological and physiochemical properties. The goal functions of the hyperparameter optimization were chosen as the mean square error (MSE) and accuracy for the regressor and classifier, respectively. The Hyperopt module was used with default settings, with the maximum number of evaluations set at 100. Training was performed using 90% of the available data, whereas the remaining 10% of data were used as a test set for final quality estimation.

### 3.2 Androgen receptor

**FIGURE 2:**
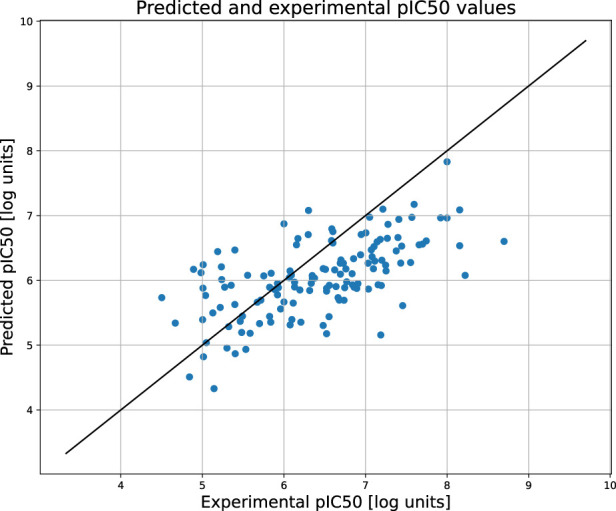
The scatter plot showing the relation between the predicted and experimental pIC50 values of androgen receptor. The predictions were obtained for the held out test set.

**FIGURE 3:**
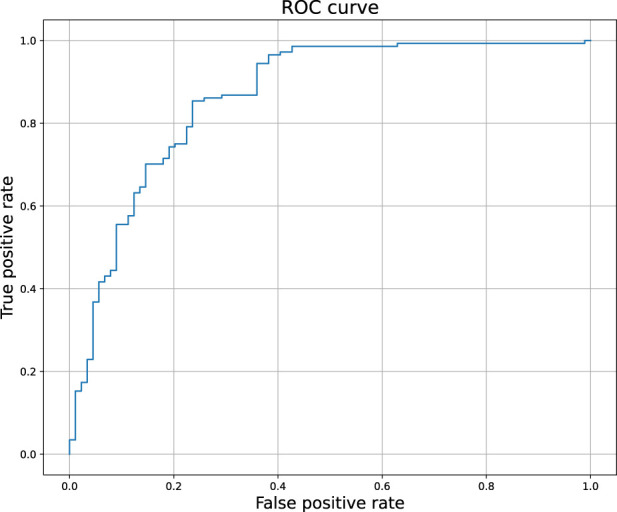
The ROC curve of the binary classifier calculated for androgen receptor. The predictions were obtained for the held out test set.

**FIGURE 4:**
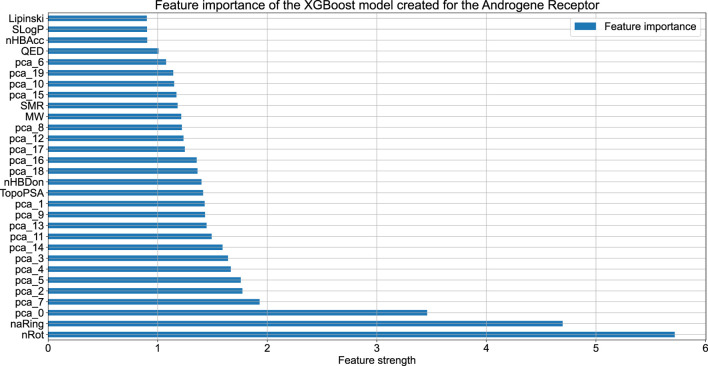
The feature importance plot obtained for the XGBoost model trained on the AR data. The plot contains the information about the 40 features having the highest influence on model predictions.

Figure 2 compares the experimental and predicted values calculated for the molecules from the test set containing 158 compounds. Approximately 80% of these molecules were predicted within a range of 1.0 log unit, and the average MSE for the entire test set was 0.61 log unit, which reflects the common predictive strength of the QSAR models. The predictive capabilities of the classification models, calculated using the hold-out test set, are listed in Table 1. The classifier reached a satisfactory level of accuracy of 82% with reasonable levels of both sensitivity and specificity. The ROC curves presented in Figure 3, and the relatively high values of the area under this curve, i.e. 0.81, clearly demonstrate the presence of a predictive signal in the data as well as the ability of the tool to extract this signal. It is worth mentioning that although the discussed models were prepared mainly for presentation purposes, they compared well relative to, or even outperformed, the available models. For instance, the accuracy, sensitivity, and specificity of the AR model were 82%, 85.4%, and 76.4%, respectively, which can be compared to the Random Forest model with 73%, 72%, and 72% as described in Piir et al. (2021). As an additional feature supporting the Machine Learning model interpretability, we delivered the feature importance capability. It is available for decision-tree-based models and provides quantitative insights into the strength of the features involved in model creation. As an example, Figure 4 shows the most influential features with the strongest contributions to the predictions for the XGBoost classifier developed for AR. The most important feature is “nRot,” which represents the number of rotatable bonds. This molecular descriptor reflects the compound flexibility, which is important from the perspective of ligand binding to the binding pocket of the receptor. Molecular descriptors and the PCA features derived from molecular fingerprints were among the 40 most important features, reflecting the topological aspects of the molecule. All configuration files and data are available in the associated GitHub repository.

**TABLE 1:** Predictive capabilities of the androgen and pregnane X receptors classification model. The results were obtained with the held out test set representing 10% of entire available data.

Measure	Value for AR [%]	Value for PXR [%]
Accuracy	82.0	82.4
Precision	85.4	82.9
Recall/Sensitivity	85.4	82.9
Specificity	76.4	80.0
F1-score	85.4	82.9
ROCAUC	80.9	82.4
Average precision	82.0	77.6
Matthew coefficient	61.8	64.8

### 3.3 Pregnane X receptor

**FIGURE 5:**
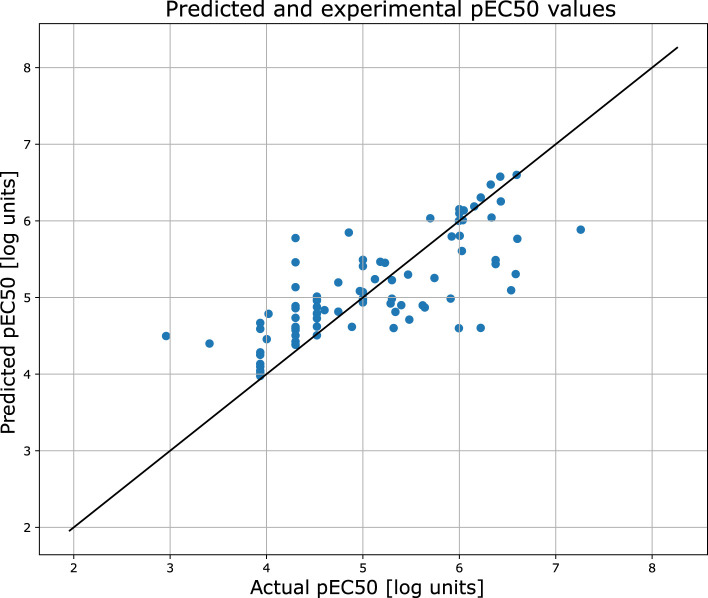
The scatter plot showing the relation between the predicted and experimental pEC50 values of the pregnane X receptror. The predictions were obtained for the held out test set.

**FIGURE 6:**
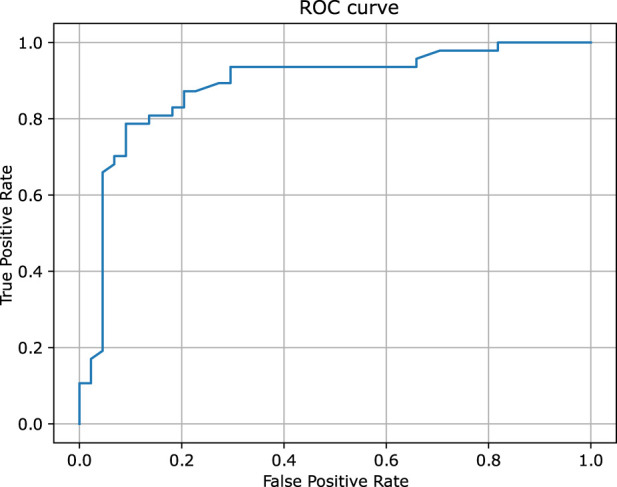
The ROC curve of the binary classifier calculated for pregnane X receptor. The predictions were obtained for the held out test set.

Similarly to the previous case, Figure 5 provides a detailed comparison between the experimental and predicted values calculated for the test set molecules associated with the PXR receptor containing 112 compounds. This figure highlights that the vast majority of these molecules - over 83% - were predicted with relatively high degree of accuracy, falling within a range of 1.0 log unit of the experimental values. This level of precision reflects the expected and consistent performance of QSAR models, reinforcing their reliability in this context. The predictive capabilities of the classification models, which were rigorously evaluated using the hold-out test set, are summarized in Table 1. The classifiers demonstrated a commendable level of efficiency, achieving sensitivity and specificity rates of 83%, indicating that the models are equally proficient at identifying both true positives and true negatives. Additionally, the model achieved an overall accuracy of 82%, further underscoring the robustness in predicting the biological activity of molecules within the PXR receptor dataset. Moreover, the ROC curve presented in Figure 6, along with the relatively high area under the curve (AUC) value of 0.82, provides evidence of a predictive signal within the data. This high AUC value not only confirms the presence of a meaningful relationship between the molecular descriptors and biological activity but also attests to the tool’s effectiveness in capturing and utilizing this signal to make accurate predictions. Overall, these results affirm the model’s practical utility and its potential for application in drug discovery and other related fields. All configuration files and data are available in the associated GitHub repository.

## 4 Summary

Here, we present a Python module called QSPRmodeler, a tool dedicated to the creation of binary classifiers and regression predictive models oriented toward predicting the biological and molecular properties of molecules. The tool utilizes the Python libraries RDKit and Mordred available from the cheminformatics site and a set of popular machine learning libraries to solve predictive analytics problems (extreme gradient boosting (XGBoost) Chen and Guestrin (2016), artificial neural networks in the form of multilayer perceptrons Bishop (1995), support vector machines Cortes and Vapnik (1995); Vapnik (1998), random forests Ho (1995), ridges Hoerl and Kennard (1970), and bagging models). The user input is limited to providing the data in an expected manner, creating the configuration files, and managing the data transformation and hyperparameter optimization. The proposed solution implements well-established machine learning practices in the context of molecules. The capabilities of the QSPRmodeler were illustrated using an exemplary application to human androgen and pregnane X receptors based on publicly available data. The resulting regression and classification models exhibited predictive capabilities and could be easily applied to various custom workflows. The implementation was provided within a permissive open-source licensing model and is available in the public GitHub repository. It is worth mentioning that the proposed tool was recently applied to the virtual screening of a large database of compounds, resulting in the discovery and experimental verification of new biologically active ligands for the ROR γ receptor Bachorz et al. (2023). As potential development avenues, we now see the inclusion of more capabilities supporting the Machine Learning model interpretability available in the Dalex library Baniecki et al. (2021), extension of the feature set to include the context of the receptor Li et al. (2024), and possibly the incorporation of complex network processing tasks that reduce the prediction error from the perspective of clustering Hu et al. (2022).

## Data Availability

The datasets presented in this study can be found in online repositories. The names of the repository/repositories and accession number(s) can be found below: https://github.com/rafalbachorz/qsprmodeler.
